# SRAM Cell Design Challenges in Modern Deep Sub-Micron Technologies: An Overview

**DOI:** 10.3390/mi13081332

**Published:** 2022-08-17

**Authors:** Waqas Gul, Maitham Shams, Dhamin Al-Khalili

**Affiliations:** Department of Electronics, Carleton University, 1125 Colonel Bay Drive, Ottawa, ON K1S 5B6, Canada

**Keywords:** 6T-SAM, low power, leakage current, process variations, soft errors, fault-tolerant, reliability, multi-threshold, noise margins, assist circuits

## Abstract

Microprocessors use static random-access memory (SRAM) cells in the cache memory design. As a part of the central computing component, their performance is critical. Modern system-on-chips (SoC) escalate performance pressure because only 10–15% of the transistors accounts for logic, while the remaining transistors are for the cache memory. Moreover, modern implantable, portable and wearable electronic devices rely on artificial intelligence (AI), demanding an efficient and reliable SRAM design for compute-in-memory (CIM). For performance benchmark achievements, maintaining reliability is a major concern in recent technological nodes. Specifically, battery-operated applications utilize low-supply voltages, putting the SRAM cell’s stability at risk. In modern devices, the off-state current of a transistor is becoming comparable to the on-state current. On the other hand, process variations change the transistor design parameters and eventually compromise design integrity. Furthermore, sensitive information processing, environmental conditions and charge emission from IC packaging materials undermine the SRAM cell’s reliability. FinFET-SRAMs, with aggressive scaling, have taken operation to the limit, where a minute anomaly can cause failure. This article comprehensively reviews prominent challenges to the SRAM cell design after classifying them into five distinct categories. Each category explains underlying mathematical relations followed by viable solutions.

## 1. Introduction

As an indispensable part of a computing system, memory dominates the semiconductor industry. According to the world semiconductor trade statistics (WSTS), memory held 27% (USD117 bn) and 28% (USD154 bn) of the total semiconductor industry market share in 2020 and 2021, respectively. By the end of 2024, the semiconductor memory market is expected to surpass USD730 bn [1]. The ever-increasing demand for fast data processing necessitates memory integration within the processor in contemporary artificial intelligence (AI) and internet-of-things (IoT) capable edge devices. Machine learning (ML) is in dire need of such devices to perform compute-in-memory (CIM) for the energy and performance-efficient algorithms implementations [2,3].

**Figure 1:**
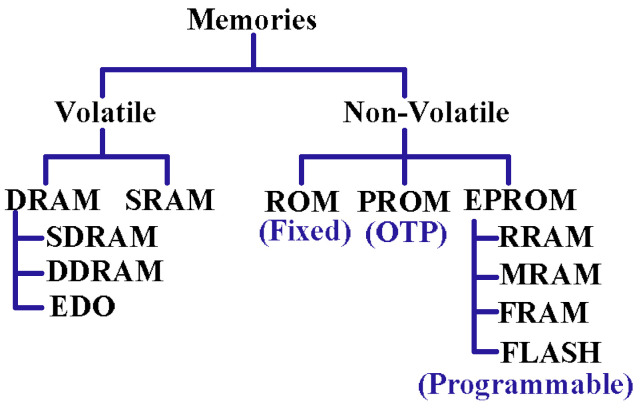
Computer memory classifications based on data retention.

Memory holds data either temporarily or permanently while processing. Two important parameters, access time and data retention, determine a memory hierarchy; faster memory placement will be closer to the processing unit. Figure 1 shows memory classifications. Several emerging nonvolatile memory cells, such as MRAM, FRAM, RRAM, PCM-RAM and FLASH, are appealing because of their improved retention time, density and performance [4,5,6,7]. However, lower latency and push-rule-based manufacturing [8] have made the SRAM cell a suitable choice for cache memory.

Technological scaling has aggressively improved SRAM performance and density. Currently, a modern SoC contains about 90% of the transistors that account for the memory [9]. However, technological scaling is also rendering a confluence of challenges. Short channel effects (SCE) [10] affect performance and enhance the leakage current. Consequently, a transistor structural shift from planer to 3D-CMOS [11] and silicon on insulator (SOI) [12] technologies mitigates the SCE and junction leakage. A FinFET-SRAM cell provides better control over the conduction channel. Thus, FinFET is inevitable in modern deep-submicron nodes. Nevertheless, the lower supply voltage poses severe threats to the SRAM cell stability. The leakage current is similarly accelerating the power budget [13]. Furthermore, variations in the threshold voltage (V_th_) have made non-erroneous SRAM cell operation alarming [14]. The reliability is a direct implication of process variations [15]. Besides the aforementioned issues, SRAM soft errors and data security are emerging areas [16]. Researchers have proposed many solutions to overcome these issues as a tradeoff for performance parameters [17,18,19]. Additionally, recent trends in CIM need reliable SRAM performance when multiple memory locations are simultaneously accessed. Therefore, a comprehensive review as a guideline for SRAM limitations and state-of-the-art solutions is essential.

**Figure 2:**
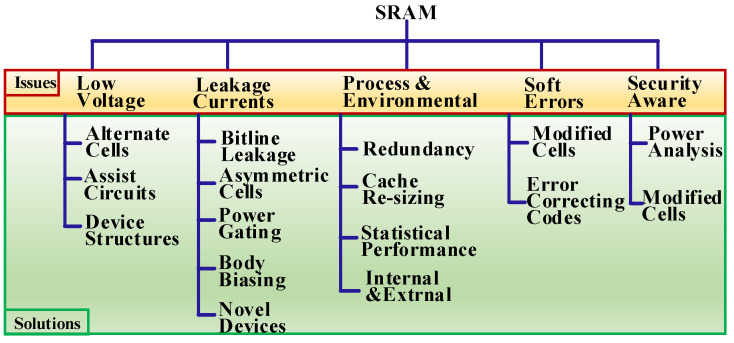
Overview of the SRAM challenges and potential solutions.

This article reviews SRAM cell design obstacles and workarounds (Figure 2). Section 2 introduces the conventional 6T-SRAM cell architecture and associated performance measurement parameters. Section 3 presents the low-voltage operation issues and available remedies. Section 4 highlights the leakage current’s significance and minimization techniques. Next, Section 5 details process variations’ impact on SRAM cell performance. Section 6 explains soft errors’ occurrence and solutions. Then, Section 7 overviews security-aware SRAM cell designs. Section 8 discusses current and future research trends. Finally, Section 9 concludes this article.

## 2. SRAM Cell

**Figure 3:**
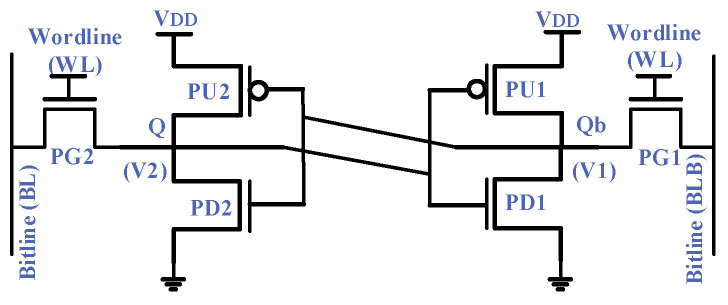
Conventional 6T-SRAM cell with: pull up (PU), pull down (PD) and pass gate (PG) transistors.

The conventional SRAM cell consists of six transistors, as shown in Figure 3. The internal nodes, Q and Qb, hold the bit value and its inverse, respectively. PMOS transistors, PU1 and PU2, pull up these nodes. Similarly, NMOS transistors, PD1 and PD2, pull down the internal nodes. The pass transistors, PG1 and PG2, provide access for the read and write operations.

An SRAM cell can operate in three modes: hold, read and write. During the hold mode, the wordline (WL) signal is low; thus, the cell keeps the internal bit value. Before a read or write operation, an initial conditioning circuit pre-charges the bitlines (BL and BLB). During the read operation, the WL signal activates both PG transistors, providing access to the stored value. One of the BLs is consequently discharged through its respective PG and PD transistors, while the other BL remains high. In this way, the SRAM cell puts the stored bit and its inverse on the BLs. To discharge a BL, the corresponding PD transistor must be stronger than its respective PG transistor, known as read stability. Equation (1) shows the relative strength of these transistors. CR values should be greater than one to ensure the read operation. Since a BL connects numerous cells, it has a high capacitive value. Accordingly, discharging takes a considerable time. Hence, a sense amplifier is used to magnify the small differential voltage between the BLs and transfers the bit value to the external circuitry.

(1) $$\mathrm{Cell}\;\mathrm{Ratio}\;\left(\mathrm{CR}\right)=\frac{{\left(W/L\right)}_{\mathrm{PD}1}}{{\left(W/L\right)}_{\mathrm{PG}1}}=\frac{{\left(W/L\right)}_{\mathrm{PD}2}}{{\left(W/L\right)}_{\mathrm{PG}2}}$$

During the write operation, a strong write driver pulls one of the BLs down depending on which value is to be written onto the cell. The WL assertion takes time, as the increased design density has put more capacitance on it. The BL Instantly pulls the storage node down via a PG transistor, but a PU transistor opposes it. This imposes a constraint known as writability. The PG transistor must be stronger than the corresponding PU transistor to copy the BL value into the SRAM cell. Equation (2) shows that the value of PR should be less than one for the write operation.

(2) $$\mathrm{Pull}\;\mathrm{Ratio}\;\left(\mathrm{PR}\right)=\frac{{\left(W/L\right)}_{\mathrm{PU}1}}{{\left(W/L\right)}_{\mathrm{PG}1}}=\frac{{\left(W/L\right)}_{\mathrm{PU}2}}{{\left(W/L\right)}_{\mathrm{PG}2}}$$

The noise tolerance level, without upsetting the undergoing operation, defines the noise margin for that particular operation. The cell and pull ratios affect the read and write noise margins, respectively. Stronger PD and weaker PG transistors ensure a higher read margin, whereas stronger PG and weaker PU transistors improve the write margin. This conflicting device sizing results in a tradeoff between the read and write noise margins. In FinFET technology, the device sizing is more challenging, as transistors’ width, represented by the number of fins, is quantized.

To measure static noise margins, the butterfly curve plot is used [20]. Since the butterfly curve method is incapable of fast and automated measurements, industrial designers rely on N-curves [21]. In N-curves, supply-read retention voltage (SRRV) and wordline-read retention voltage (WRRV) render the read noise margin measurements. For the write noise margin, bitline-write trip voltage (BWTV) and wordline-write trip voltage (WWTV) furnish the write noise margin measurements [22]. Table 1 mentions details of key performance evaluation parameters of an SRAM cell.

**Figure 4:**
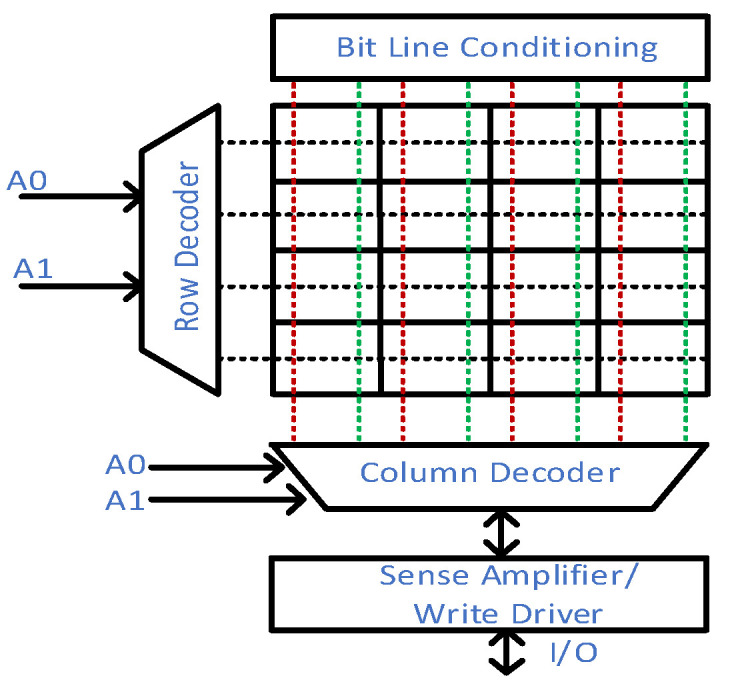
A 4 × 4 (16 bits) SRAM architecture for a complete operation as memory cell.

The aforementioned conventional 6T-SRAM cell necessitates the peripheral circuitry for operational procedures. Figure 4 presents the main peripheral components: row and column decoders, sense amplifiers, bitline conditioning and write drivers. The external components’ performance plays a crucial role in the overall memory design. For example, amortization reduces the number of sense amplifiers required for an SRAM cell array design [22]. Hence, an SRAM array achieves efficiency in design density and power consumption.

## 3. Low Voltage Operation

Dynamic power contributes a major constituent in the total power consumption of a digital circuit. The integrants’ composition (${\alpha \mathrm{CfV}}_{\mathrm{DD}}^{2})$ is switching activity (α), switching frequency (f), node capacitance (C) and supply voltage ($V_{\mathrm{DD}}$). The supply voltage is a squared term that indicates the highest contribution. The tuning of parameters α and f affects the SRAM cell performance but increases the power budget. Technology advancement reduces capacitances (C), but the improved design density diminishes overall returns.

**Figure 5:**
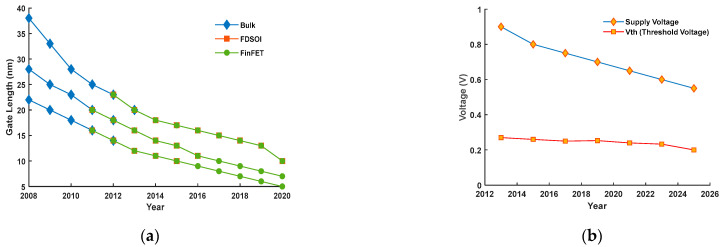
(**a**) Gate length evolution of application-specific circuit design for low standby power (LSTP), low operational power (LOP) and high performance (HP). (**b**) Supply voltage and threshold voltage scaling decreasing the noise margin headroom. (ITRS).

Transistor downsizing leads to scale-downs in the supply voltage. T.H. Bao et al. [23] reports feature sizes down to 5 nm as workable at the commercial scale. The international roadmap for devices and systems (IRDS 2022) predicts transistor scaling down to the 0.7 nm node by 2034. However, size minimization brings forward obstacles such as degraded sub-threshold slopes (SSs) and increased drain-induced barrier lowering (DIBL) [24]. The SRAM cell design has consequently shifted from planar devices to fully depleted silicon-on-insulator (FDSOI) [25], which permits an acceptable performance, as shown in Figure 5a. In the future, sheet or gate-all-around (GAA) transistors [26] will replace the current FinFET in the SRAM cell design.

Figure 5b explains the compromise on noise margins as the supply voltage reduces. An SRAM cell can initially operate at a V_DD_ of 0.9 V and then a decreasing trend continues to 0.5 V. The threshold voltage (V_th_) could not comparatively follow this trend at the same pace.

### 3.1. Alternate Cells

To overcome the low supply voltage challenge, designers have put forward multiple SRAM cell designs [26,27,28,29,30,31]. Primarily, alternate SRAM cells aim to improve the noise margins. Enhanced noise margins ensure a particular SRAM cell has sufficient stability room at a decreased supply voltage.

**Figure 6:**
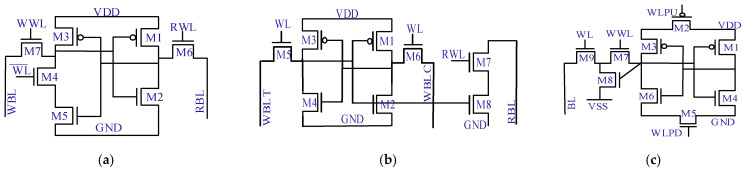

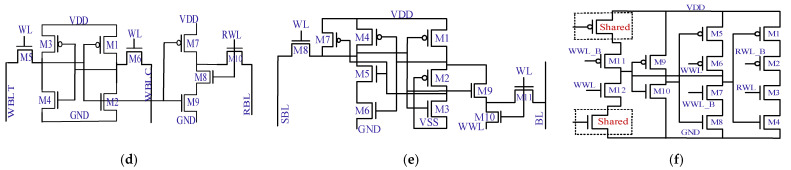
Alternate SRAM cell designs for low voltage operation using feedback break, isolated port, power-gating, buffered values, read or write independency and concurrent read or write strategies. (**a**) 7T-SRAM cell [26], (**b**) 8T-SRAM cell [27] (**c**) 9T-SRAM cell [28], (**d**) 10T-SRAM cell [29], (**e**) 11T-SRAM cell [30], (**f**) 12T-SRAM cell [31].

Figure 6 highlights different SRAM cells and associated control signals. Without loss of generality, the basic operation is similar in essence, as explained in Section 2. A 7T-SRAM cell, as seen in Figure 6a, has a single read-bitline (RBL) and read-wordline (RWL) for the read operation. The newly added transistor M4 breaks the back-to-back inverters’ feedback through the WL signal and improves the write noise margin. An 8T-SRAM cell, as seen in Figure 6b, separates the read-port (M7 and M8) to avoid device size conflict and thus, this cell can independently tune the read noise margin. Furthermore, a 9T-SRAM cell, as seen in Figure 6c, has a separate read and write port (M7, M8 and M9). Additionally, this cell has wordline pull-up (WLPU) and wordline pull-down (WLPD) transistors for the power gating to save power. Next, 10T-SRAM cells, as seen in Figure 6d, manifest the separate read and write port concepts as the same as 8T-SRAM cells. But the M7, M8 and M9 transistors’ buffer provides strong logic for the 0 or 1 value. The addition of M2, M5 and M7, as seen in Figure 6e, is an alternate way to furnish the strong logic value. Transistors (M9 and M10) in an 11T-SRAM cell remove interdependency between the read and write noise margins. Tri-state buffers for the read and write operations in a 12T-SRAM cell, as seen in Figure 6f, improve the read- and write-energy per cycle while the read or write operation is in progress.

Table 2 reports the performance parameters analysis among Figure 6 cells. It is noted that the proposed SRAM-design cells, with the extra transistors, enhance the area and put stringent requirements on the layout regularity. Although the alternate cell designs show better read and write ability as compared with their counterparts, this is at the expense of more control signals and increased area. Adaptability and flexibility to other components determine the overall efficiency of the specific SRAM cell design.

### 3.2. Assist-Circuits

A lower supply voltage improves dynamic power but makes read and write operations challenging. Therefore, assist-circuits ensure an SRAM cell operations’ reliability. Two circuit-level choices for a low voltage operation are the use of assist-circuits and adaptation to alternate SRAM cells. Both options have tradeoffs at the cost of extra hardware and control signals. In FinFET technologies, the 6T-SRAM cell utilization alongside assist-circitry is inescapable to guarantee the high design density.

As illustrated in Figure 3, the conventional 6T-SRAM cell is connected to three types of control signals: voltage supplies (V_DD_ and G_ND_), BLs and WLs. An assist-circuit raises or lowers the signal voltage level as per the operational mode. Table 3 shows eight different possible combinations of assist schemes [32].

In a negative bitline (NBL), a coupling capacitor (CC) generates the negative voltage on a BL to aid in flipping the cell value. However, the charging and discharging of CC as an additional component raises the overall power consumption [32]. Recently, TSMC proposed the design technology and co-optimization (DTCO) [33] for the generation of CC using the BL-length adjustment, optimizing the operational voltage by 300 mV at the 5 nm node. Next, the suppressed bitline (SBL), instead of pre-charging BLs to the V_DD_ level, discharges BLs to the intermediate voltage. The lower voltage level discharge ascertains read operation assistance. Furthermore, wordline over-drive (WLOD) needs to use a charge pump and biasing circuitry to achieve a higher voltage level. The PG transistors become stronger and assist in the write operation. However, the gate oxide comes under stress because of the high voltage level. Opposite to WLOD, wordline under-drive (WLUD) assists by weakening the PG transistors (WL voltage lowering). For boosting V_DD_, a column MUX helps to choose the desired level. Extra power line grids and pads incur more area and delay penalty. Enhanced V_DD_ aids in putting the internal node value onto BLs faster, whereas the V_DD_ lowering helps in flipping the stored value of the SRAM cell. PMOS devices along biasing current circuits achieve adjustments in the dynamic supply voltage. The last two schemes in Table 3 raise or lower the ground level, but the G_ND_ rail sharing among all of the SRAM cells is challenging.

Researchers have also used combinations of multiple assist schemes such as SBL and NBL; and dual-transient wordline (DTWL) [34]. The underlying strategy is to segregate each control signal (WL, BL, V_DD_ and G_ND_) and then apply an increased or decreased voltage level on each of these signals to perform low power read and write operations [35,36]. Transient voltage collapse (TVC) (V_DD_ lowering) [37,38], stepped WL (WLUD) [37] and dual write driver [39] assist low voltage operations. Before target application information, low power (LP), high density (HD) or high performance (HP) enables circuit designers to set the transistors’ strength to effectively achieve the design’s goal.

### 3.3. Device Structure

**Figure 7:**
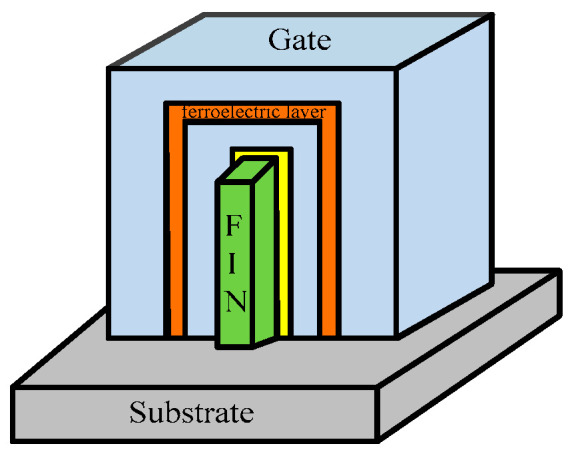
Negative capacitance-based FinFET with ferroelectric layer as dielectric [40].

**Figure 8:**
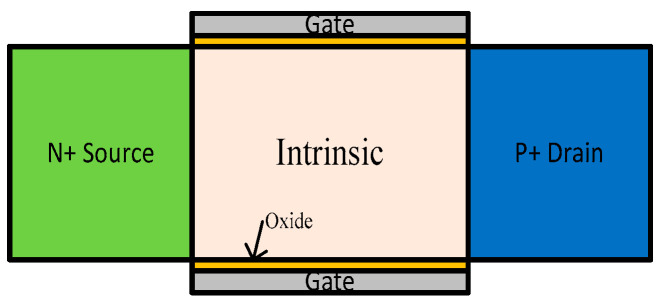
TFET structure: a cross-section showing doping levels [41].

Innovation in device structure is another option to operate SRAM cells at a lower voltage. Two classes of device structure modifications are available: the back end of line (BEOL) and front end of line (FEOL). Figure 7 and Figure 8 show one structure from each class as a representative model.

Negative capacitance (NC) [40] in FinFET-SRAM cells provides better noise margins. Insertion of the ferroelectric material between two gate metal layers, as shown in Figure 7, creates NC. The applied gate voltage polarizes the ferroelectric dielectric that amplifies the gate voltage at the second gate metal layer underneath the dielectric material. This improves the I_ON_/I_OFF_ ratio, leading to better SS and DIBL. The thickness and composition of the dielectric material are a tradeoff with the read and write performance.

The second type of device structure is illustrated in Figure 8. It is a tunnel FinFET (TFET) with both terminals, source and drain, joined through an intrinsic channel [41] and has a doping of opposite polarities. Such transformation improves the sub-threshold factor (KT/q) and gate-work function to support the low voltage operation down to 0.3 V.

## 4. Leakage Current

Technological node scaling has improved the design density; subsequently, a greater number of transistors on the same IC area result in a considerable increase in leakage power. Now leakage current constitutes a significant portion of the overall power budget.

A modern transistor exhibits three leakage currents: the tunneling current between conduction channel and gate, PN junction or body leakage current between source and drain and body interface, and the sub-threshold conduction current between the source and drain under sub-threshold voltages due to DIBL and GIDL [10]. However, the body leakage current is no longer a serious concern in modern technologies, as SOI [11] has reduced the junction leakage magnitude. Considering Figure 3, assume the SRAM cell stores ‘1’, meaning the node Q is at a logic of ‘1’, whereas the node Qb is at a logic of ‘0’, and both BLs are pre-charged. Under this condition, transistors (PU1, PG1 and PD2) suffer from sub-threshold leakage. Three transistors (PU2, PG2 and PD1) will face the sub-threshold leakage in case an internal node stores the ‘0’ value, thus confirming unavoidable leakage in either case.

### 4.1. Bitline Leakage

Bitlines leak current through PG transistors. An approach to reduce this leakage is by the BLs’ initial condition. The assist-circuits presented in Section 3, such as NBL and SBL, reduce the leakage as well [42]. In both assist schemes, the voltage on BL is less than V_DD_; hence, the leakage current subsides. Likewise, the WLUD-assist scheme reduces the leakage current from the internal nodes towards BLs. V_DD_ lowering similarly decreases both power consumption components, i.e., dynamic and leakage. The assist schemes in Table 3 minimize leakage currents except in the lowering of G_ND_, boosting of the V_DD_ and WLOD schemes. These schemes widen the rail-to-rail potential difference, eventually increasing the leakage current.

### 4.2. Asymmetric Cells

**Figure 9:**
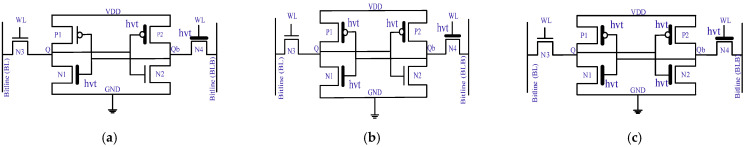
Asymmetrical 6T SRAM cells for leakage reduction using preferential state and multi-threshold voltage [43]. (**a**) Asymmetric: ‘1’ as preferential state; (**b**) asymmetric: ‘0’ as preferential state; (**c**) asymmetric leakage optimization.

**Figure 10:**
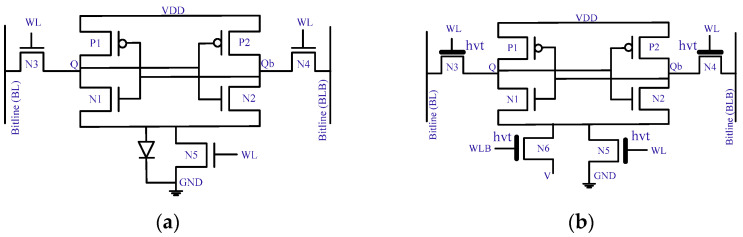
Leakage control mechanisms for SRAM cells using internal components and external control signals for raised ground levels [45,46]. (**a**) SRAM cell with virtual ground; (**b**) asymmetric SRAM cell with virtual ground.

The multi-threshold SRAM cell design minimizes the leakage current by choosing ‘0’ or ‘1’ as the preferential state. Each logic state associated with a leakage current has already been explained above (Section 4). Leaky transistors are set to a high threshold value (hvt) to reduce the leakage [43]. This approach, as seen in Figure 9a, reduces the leakage about 70 times by choosing ‘1’ as its preferential state, but at the cost of a degraded read ability. The SRAM cell in Figure 9b provides 1.6 and 70 times less leakage current for selecting ‘1’ and ‘0’, respectively, as preferential states. This asymmetry causes an increase in write delay. Next, the SRAM cell in Figure 10c reduces the leakage current about seven times for the ‘0’ state but compromises the read and write noise margins.

Ghasem et al. [44] proposed the SRAM cell size variation in combination with multi-threshold voltage designs at the architectural level. SRAM cells in the same row exhibit multiple delays, i.e., cells farther from the row driver have more delays and vice versa. A row decoder drives longer wires and more capacitance for the SRAM cells placed towards the end of a row. Hence, the speed of cells located towards the end of the row is slower. The use of multi-V_th_ has improved not only the delay but also the leakage of the overall SRAM array.

### 4.3. Alternate SRAM Cells

The conventional 6T-SRAM cell suffers from stability issues, especially in FinFET technology. Researchers have explored more than 6T-SRAM cells to reduce the leakage current. We have already illustrated some configurations in Figure 6. Table 2 provides details of each SRAM cell leakage power. The 7T-SRAM and 8T-SRAM cells show comparable leakage power to the 6T cell since transistor stacking reduces the leakage current. In the case of 11T-SRAM cells, the leakage value significantly reduces due to transistor stacking in both feedback inverters.

### 4.4. Power Control

Additional transistors along the control signals in an SRAM cell can cut off the power. One approach is to power-gate a few transistors in a cell, as shown in Figure 6c. Two other mechanisms are controlling the ground node through internal components [45] and external signals [46]. Architectural level power gating reduces leakage drastically [47].

Figure 10a demonstrates the first mechanism; the WL signal controls the connection to the ground through transistor N5, and the internal diode prevents internal node flipping by raising the ground node level. Figure 10b, as representative of the second mechanism, provides a stringent grip over the leakage as compared with Figure 10a. High V_th_ and ground-level control transistors manage the leakage current but cost extra signals.

### 4.5. Body Biasing

**Figure 11:**
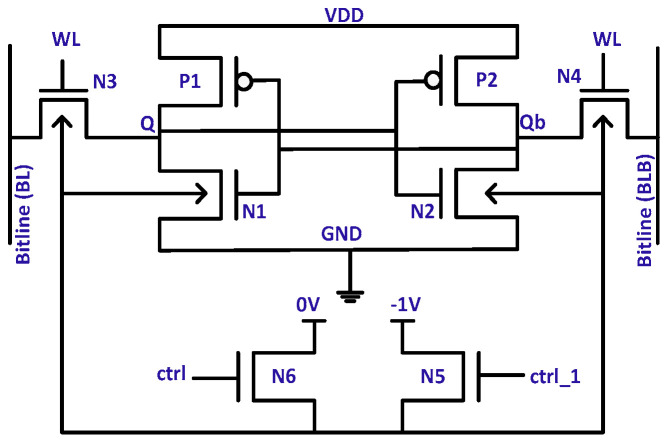
SRAM cell with body biasing control circuit [48].

To improve the performance and leakage currents, two prevalent body biasing mechanisms are forward body biasing (FBB) and reverse body biasing (RBB). The SRAM cell [48] shown in Figure 11 exploits both techniques through N5 and N6 transistors with the external control signal ‘ctrl.’ Two transistors, N5 and N6, select voltage levels of ‘-1′ and ‘0’, respectively, to utilize RBB. In modern FinFET technology, SOI has eliminated body biasing. However, FinFET-independent gates (IG) biasing IGFET [49] offers a modulation of the threshold voltage to subside the leakage current.

### 4.6. Novel Devices

**Figure 12:**
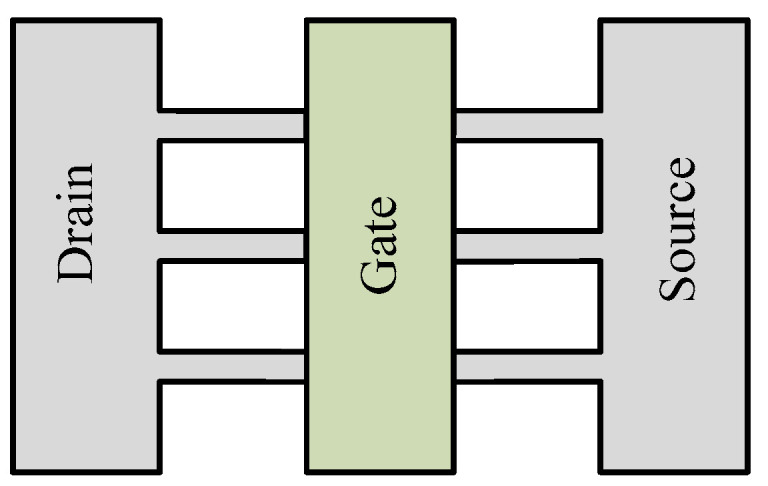
Graphene nano-ribbon structure of FinFET [50].

First, graphene nano-ribbon FET (GNRFET) holds excellent conducting properties [50] due to un-doped GNR channels underneath the gate (reduces leakage) and highly doped GNR channels between the gate and source and drain terminals. Figure 12 (FEOL) shows the ribbon-shape structure of the GNRFET.

**Figure 13:**
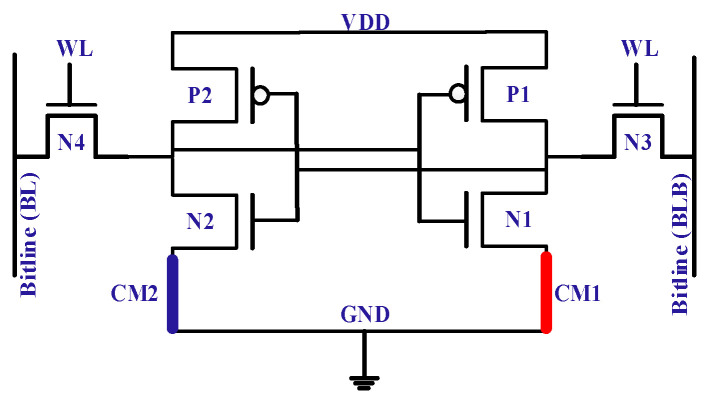
Correlated material-based 6T-SRAM cell [51].

**Figure 14:**
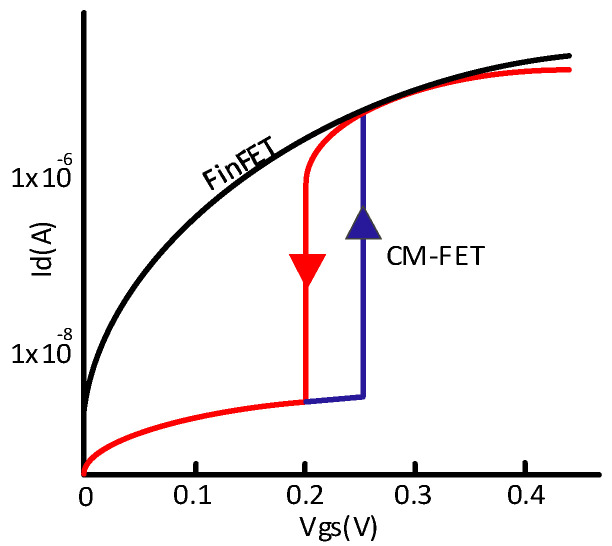
HFET &FinFET transition between ON and OFF states [51].

Another device structure [51], shown in Figure 13, shows improved off-state performance for the correlated-material (CM) (or hyper FET) SRAM cell. CM-FET performs the transition from the insulator to metal (ITM) and then back from the metal to insulator (MTI). The hysteresis curve in Figure 14 demonstrates the transitions of ITM and MTI from 0.20 V to 0.27 V. Th on-current of CM-FET and FinFET are comparable; however, the off-current of CM-FET is substantially lower.

## 5. Process and Environmental Variations

### 5.1. Process Variations

In modern technology, variations in parameters characterizing the device performance are comparable with their nominal values. Some device instances show different performances, and they sometimes are unable to meet specifications. Nevertheless, a degree of uncertainty in performance exists. FinFET smaller size affects manufacturing yield owing to the process variations.

**Figure 15:**
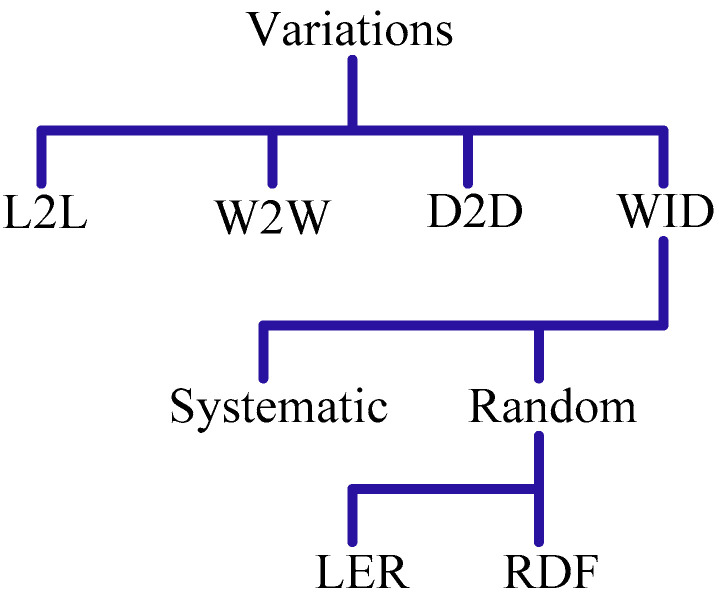
Process variations in the deep sub-Micron and FinFETs [8,52].

Figure 15 presents categories of process variations: lot-to-lot (L2L), wafer-to-wafer (W2W), die-to-die (D2D) and inter- or within-die (WID) variations. The L2L and W2W variations prevail within different lots of the cylindrical silicon boules and circular silicides of the same boule, respectively. The L2L and W2W effects on the circuit performance are generally minute; therefore, they are ignored quite often. However, D2D variations influence device parameters on different dies belonging to the same wafer.

The systematic (WID) variations [52] are usually caused by some anomaly in the system during the mass production process, while the random (WID) variations are a direct result of the random behaviors in recent technological nodes. These variations are inscrutable and require statistical distribution for their characterization. First, dopant atoms give rise to the phenomenon of random dopant fluctuation (RDF). Second, the gate patterning has not been smooth and straight in modern feature-length sizes, referred to as the line edge roughness (LER). Equation (3) shows variations in the threshold voltage, where q is the charge; $\varepsilon_{\mathrm{si}}$ and $\varepsilon_{\mathrm{ox}}$ are the permittivity values of silicon and gate oxide, respectively; $N_{a}$ is the dopant concentration; $\Phi_{B}$ is the energy level of inter-potential bands; $t_{\mathrm{ox}}$ is the gate oxide thickness; W and L are the channel width and length, respectively.

(3) $$\sigma_{V_{\mathrm{th}}}=\left(\sqrt[{4}]{2q^{3}\varepsilon_{\mathrm{si}}N_{a}\Phi_{B}}\right)\frac{t_{\mathrm{ox}}}{\varepsilon_{\mathrm{ox}}}\frac{1}{\sqrt{3\mathrm{WL}}}$$

**Figure 16:**
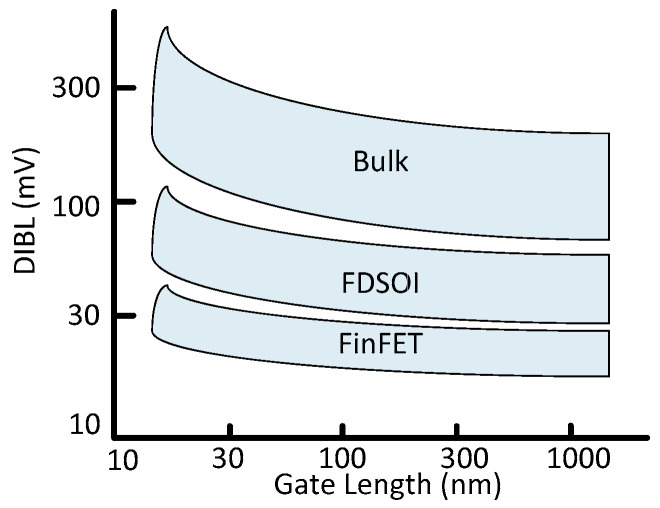
DIBL in bulk fully depleted silicon on insulator (FDSOI) and FinFET [54].

FinFET technology with moderate doping inside the channel shows fewer V_th_ variations [53]. But Fin geometrical dimensions and quantized natures still show the V_th_ variations for the SRAM cell design. Figure 16 [54] shows variations in DIBL, whose sequel is the V_th_ variations. Equation (4) describes the interdependency of DIBL and V_th_. The parameter $V_{\mathrm{th}\infty }$ represents the intended value of the threshold voltage, whereas $V_{\mathrm{th}}$ is the actual value.

(4) $$V_{\mathrm{th}}=V_{\mathrm{th}\infty }-\mathrm{SCE}-\mathrm{DIBL}$$

Process variations pose challenges to reduced noise margins, stability and malfunctioning. Solutions such as assist-schemes, alternate cell designs and novel device structures are not sufficient. Hence, architectural level remedies substitute the faulty SRAM cells to ensure a workable memory array.

A. Redundant rows and columns: The introduction of extra SRAM cells avoids failure because of the process variations. Faulty cells can be replaced in two ways. One way is to switch from a failed SRAM sub-array to a redundant sub-array, irrespective of the number of defective rows and columns. Otherwise, an extra row and column of cells can substitute only failed rows and columns. However, the re-routing of redundant SRAM cells needs complex control signals and address generations that boost the area overhead, the faulty cells recovery yield, and the switching timing penalty.

**Figure 17:**
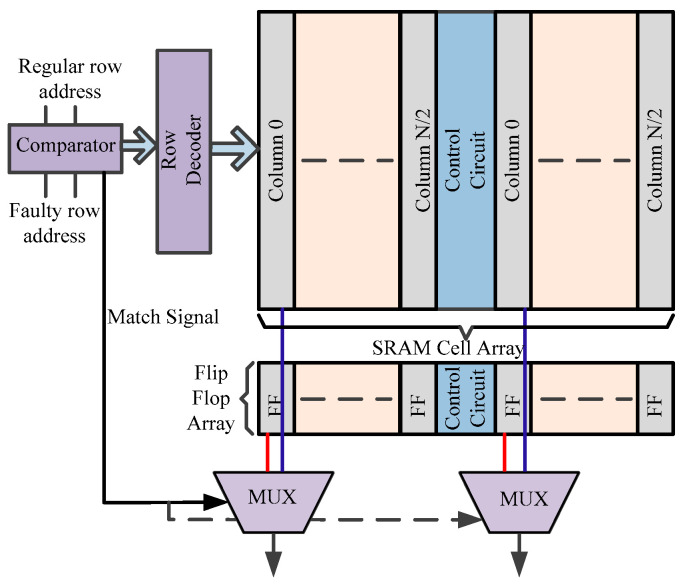
Architecture of row redundancy scheme using flip-flops [55].

The technique proposed in [55] uses a master-slave latch instead of extra rows of SRAM cells. Figure 17 illustrates the flip-flop redundancy technique. Here, the redundant row of flip-flops, a comparator and MUX are the extra hardware components. During an operation, a comparator compares both addresses, i.e., regular and faulty. If they match, then the control signals divert the path to the redundant flip-flops to avoid faulty cells.

**Figure 18:**
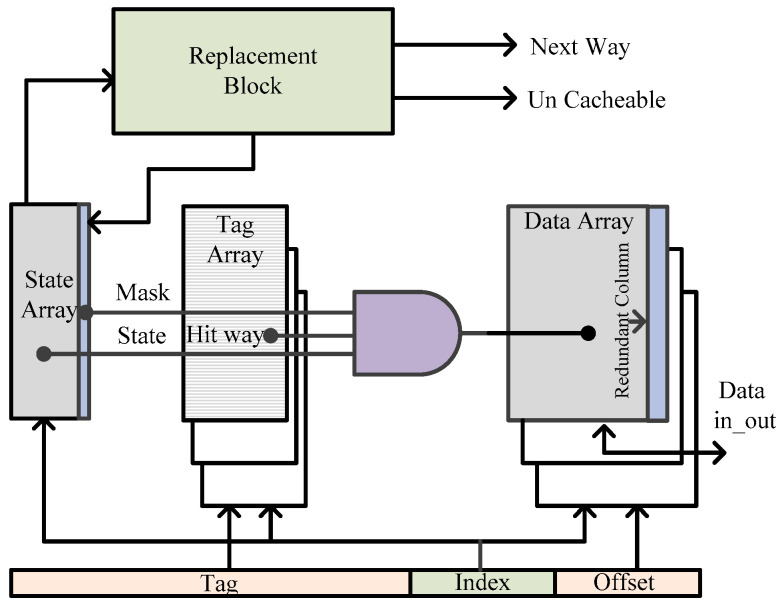
Runtime re-sizeable cache architecture [56].

B. Dynamic cache resizing: Built-in-self-tests (BISTs) together with dynamic caches bypass the faulty locations [56]. Defective-cell-avoidance improves the SRAM chip yield and tradeoffs with the performance. Figure 18 shows the architecture of a re-sizable cache. Data arrays contain a sea of SRAM cells. A tag-array-block determines the location of the SRAM data array (hit way) to be accessed, using the tag and index information. In addition, the state array validates a particular tag. The resizable architecture contains an extra masking bit in the state array, providing information on whether an accessed location is faulty or not.

Moreover, BIST architecture tests the whole data array and retrieves the faulty SRAM cells. To maintain a cache’s performance, the system immediately performs BISTs after turning on the power. Once the number of faulty cells exceeds the threshold value, the cache becomes unusable.

**Figure 19:**
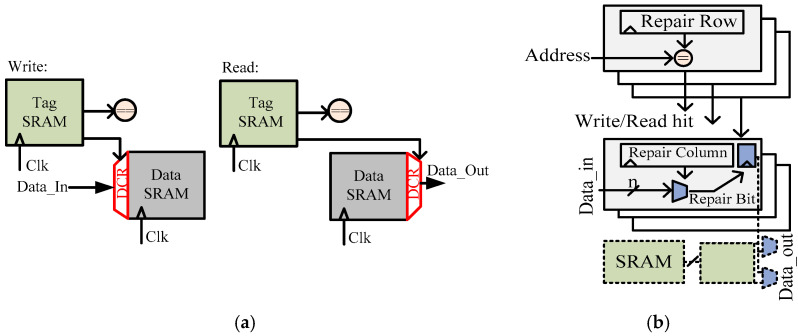
Flip-flop-based row redundancy architecture [57]. (**a**) Dynamic column redundancy (DCR); (**b**) bitline bypass (BB).

C. Reprogrammable Redundancy: The SRAM hardware run-time reconfiguration makes it robust, as it is applicable even after SRAM-chip testing. Figure 19a [57] shows dynamic column redundancy (DCR). A spare column is inserted with the SRAM’s data. A DCR contains two-way multiplexers. A memory controller dynamically assigns them according to fault occurrence in an SRAM array. Internal redundancy access (RA) re-routes the redundant column cell to the faulty location. Tag SRAM cells hold RA information and subsequently share across all lines once a fault occurs. DCR offers minimum area and timing overhead as compared with the technique where redundant rows substitute an entire faulty sub-array.

Bitline bypass (BB), shown in Figure 19b, repairs faulty SRAM cells through the redundant rows or columns of SRAM cells. When repairing is complete, row and column addresses are re-programmed to ensure the correct operation. The timing overheads to bypass faulty SRAM cells are the setup time during the write operation and the multiplexer delay in the read operation. Another simpler approach is line disabling (LD), where faulty lines of SRAM are disabled at the cost of a reduced cache size.

D. Statistical Performance Evaluation of the SRAM Cell: This unique approach discards SRAM caches showing under-performance during the testing phase. The performance evaluation against a benchmark is a fair criterion to decide a particular cache’s acceptability.

The corner-case analysis conventionally manifests an SRAM performance evaluation under extreme scenarios. Recently, increasing WID variability shows variations in the performance of PMOS and NMOS devices. Thus, uncertainty in SRAM cell performance increases. Monte Carlo (MC) simulations lay out the statistical performance with random variations. The probability of a certain event, E, is accomplished through the unknown random variable X distribution. The randomization produces a huge sample space with N number of samples. Equation (5) describes the probability of an interest event [58].

(5) $$\;\hat{P}{}_{\mathrm{MC}}=\frac{1}{N}\sum_{i=1}^{N}1\left(X_{i}\in E\right)$$

In some cases, the number of samples is effectively reduced by employing the concept of importance sampling (IS) [58]. A careful analysis provides a relationship function f between random variable X and event of interest E. Equation (6) sets forth the probability distribution through IS.

(6) $$\;\hat{P}{}_{\mathrm{IS}}=\frac{1}{N}\sum_{i=1}^{N}f\left(X_{i}\right)1\left(X_{i}\in E\right)$$

The efficacy of sampling can be improved by utilizing the algorithms such as norm-minimization [58], loop-flattening, spherical [58] and Gibbs sampling [59].

### 5.2. Environmental Variations

The temperature is a paramount factor in environmental variations. Any variation in the temperature affects the charges’ transportation in a device and accordingly impacts the circuit performance. At low temperatures, SRAM performance is better due to the high I_ON_/I_OFF_ current ratio. Increasing the design density shows more leakage current on the same footprint, which appears in the form of heat dissipation and, hence, raises the temperature. Therefore, modern technologies’ performance suffers more due to the improved design density.

Temperature control mechanisms are either internal or external to a chip. External mechanisms include heat sinks, fans and liquid nitrogen. Internal mechanisms control the temperature at the circuit or architecture level. During the circuit design, minimizing the leakage current helps in limiting the temperature rise.

The throttling mechanism [18] prohibits the temperature elevation at the architecture level, resulting from operational conditions and process variations. The throttling method continuously tracks the temperature value. As the value crosses the critical level, control circuitry lowers the operational frequency and voltage. The system restores nominal operational parameters provided that the temperature has returned to an appropriate range.

Besides the control of the supply voltage and operational frequency, temperature-aware perspective methods are (1) body biasing, (2) BL sensing and (3) WL voltage. These methods maintain the SRAM cell performance despite the temperature variations.

Body biasing voltage tuning shows improved performance despite temperature fluctuations [60]. Performance is sustained on a chip by the generation of multiple voltages and then switching to a suitable level. Another challenge is the BLs’ sensing margin reduction. The leakage current is more dominant at high temperatures because of charge scattering. In the SRAM design, the leakage current flows from BL to the access transistor. As currents flow through the BLs’, the voltage level is reduced, which is an issue in a read operation. A temperature controller tracks the leakage current and compensates for any deficient voltage level to restore the BL voltage [61]. Similarly, the WL voltage lowering [62] in a higher temperature enhances the read performance while maintaining the operational frequency. The temperature compensation circuit [61] regulates the WL voltage. Similarly, a circuit-level modification [63] where a buffer replaces the access transistor and sense amplifier demonstrates a reliable operation up to 275 °C in SOI technology.

## 6. Soft Errors

Environmental conditions can cause the emission of alpha particles, high-energy neutrons and muons from a packaging material [64]. These particles possess sufficient energy to alter the SRAM storage node. Scaling has lowered the critical charge on recent technological nodes; hence, expediting the internal state logic inversion. However, FinFET reduced geometry and drain-area exposure to the striking of high-energy particles lessens the soft errors rate [65].

Radiation-induced soft errors can either be a single event upset (SEU) or multiple events upset (MEU). Regardless, the exposed nodes of the SRAM cell collect the charge (Q). When a node charge exceeds the critical charge value, it switches a node to the opposite logic level. Equation (7) models the critical charge [66].

(7) $$Q_{\mathrm{critical}}=Q\left(1-e^{\frac{-t_{f}}{\tau }}\right)$$

The critical charge ($Q_{\mathrm{critical}}$) is the minimum charge to invert a nodal value. The charges collected depend on the fall ($t_{f}$) and rise times of the internal voltage (τ) to change the internal node state. Equation (8) explicates the number of SEUs. Apart from the critical ($Q_{\mathrm{critical}}$) and collected charges ($Q_{\mathrm{collected}}$), SEU is also dependent on the flux (Φ) and sensitive areas (A).

(8) $$N_{\mathrm{SEU}}=\Phi .{\mathrm{Ae}}^{\left(-\frac{Q_{\mathrm{critical}}}{Q_{\mathrm{collected}}}\right)}$$

Specifically, MEUs are triggered by bipolar amplification and charge sharing [67]. Bipolar amplification is the result of a bipolar transistor formation between the source and drain, with the body acting as a base. Equation (9) characterizes the number of MEUs ($N_{\mathrm{MEU}}$), which depends on the occurrence of SEUs ($N_{\mathrm{SEU}}$) and the probability of multiple upsets ($P_{\mathrm{MEU}}$).

(9) $$N_{\mathrm{MEU}}=N_{\mathrm{SEU}}.P_{\mathrm{MEU}}$$

Here in Equation (9), $P_{\mathrm{MEU}}\approx 1-Q_{\mathrm{critical}}/Q_{\mathrm{collected}}\left(x\right)$, x represents the relative distance along the direction of charge collection.

Soft error remedies can be performed at three levels: processing, cell and architecture. The processing level treatment alters the device structure and doping concentrations. These modifications either increase the critical charge or make devices less exposed to radiation. Structural innovations consequently enhance the SRAM cell tolerance against soft errors. Modern FinFET, PDSOI and FDSOI show fewer error rates due to processing level modifications [64]. The last two soft error remedies are presented below.

**Figure 20:**
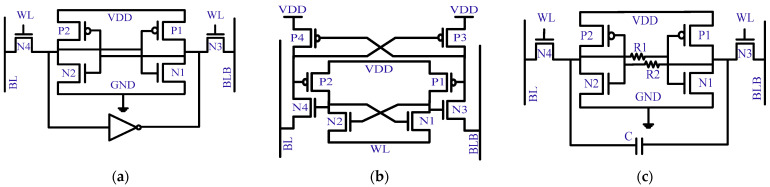
SRAM cell hardening for soft errors mitigation. (**a**) Increasing critical charge value [68]; (**b**) indirect connection to internal nodes [69]; (**c**) external components to slow down response [70].

A. Modified SRAM Cells: The SRAM cell hardening either enhances the sensitive-node critical charge or slows down the transistor response. Figure 20a shows the addition of an extra inverter in the conventional 6T-SRAM cell [68]. The newly added inverter increases the critical charge and refreshes the sensitive node value. Similarly, a fully interlocked SRAM cell cuts off a direct connection to the internal node, i.e., P1 and P2 drains, as shown in Figure 20b [69]. The nodes connected to the gates of P1 and P2 are passed down to BLs. This suppresses any external leakage and exposure to the internal nodes. Under normal circumstances, both BLs are at a logic of zero. Next is the hybrid approach, as demonstrated in Figure 20c [70]. The presence of the coupling capacitor multiplies the critical charge value, while resistors slow down the SRAM cell response. The value of coupling capacitors and resistors decides the degree of soft error tolerance. However, the addition of integrated components accelerates power exhaustion.

B. Error Correcting Codes: The simpler way to implement error correcting codes (ECC) is to add a parity bit to each of the words in an SRAM array. The parity bit is an XOR of all the bits in a word. In case a soft error alters an SRAM cell value, the parity bit value would be different than the XOR result of the bits in that specific word. But only the single soft error detection functions with parity. The basic approach followed for ECC is as follows: data bits’ encoding is followed by syndrome calculation using codes already implemented; then, the syndromes’ comparison locates the error bits. Errors correction is the last step to complete the rectification.

**Figure 21:**
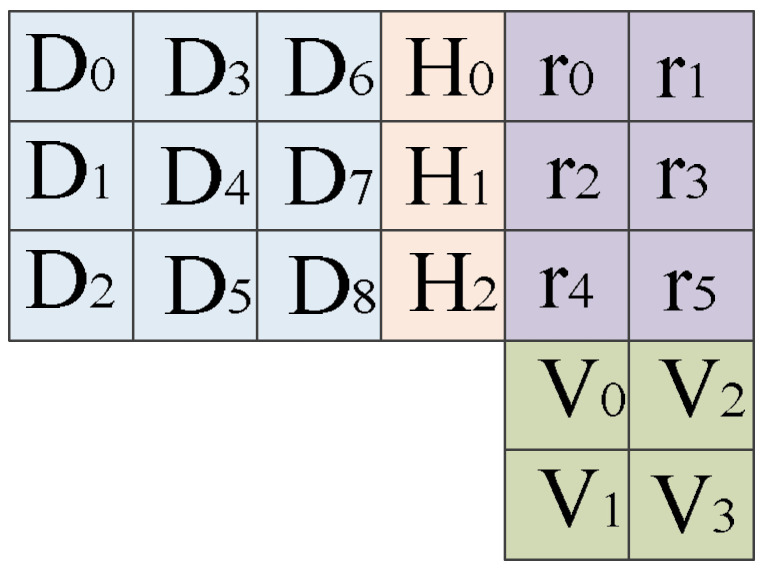
Matrix errors’ correcting codes for error detection and correction [71].

Matrix ECCs are appealing because of their low complexity in implementation. They divide an SRAM array into the matrix format at the logical level. Each horizontal row and vertical column contain the parity bits. Figure 21 shows the logical partitioning of memory using matrix codes [71]. In Figure 21, D represents data bits, H is the horizontal parity hamming bit, while r is the hidden hamming bit. At the bottom, V shows the vertical parity bit. Taking the XOR of all the data bits in a row produces a horizontal hamming parity bit. Hidden hamming (r) bit XORs alternate data bits for the same row. Vertical parity (V) bits are the result of alternate hidden hamming bits’ XOR operation. The comparison of bits r and V locates data bits containing an error. This way, a matrix code detects and corrects multiple errors in a particular SRAM block.

Likewise, column line codes (CLC) use extended hamming codes for error detection and correction [72]. In addition to parity bits, extended codes enable the detection and correction of the maximum number of errors. The number of redundant bits raises to increase the error tolerance.

However, to ameliorate the hardware redundancy, the single-error-correction double-error-detection (SEC-DED) or double-error correction (DEC) offers a solution through direct comparison [73]. This incorporation immensely reduces the overhead, whereas the cache-hit triggers error-bit detection and correction.

## 7. Security-Aware Design

As part of cache memory, an SRAM cell array might hold sensitive data in an embedded system design. Data attackers can exploit design parameters for the memory envision. Table 4 provides a summary of such data stealing techniques.

A. Data Attacks

1. Power Analysis (PA) Attack [74]: Side channel analysis can trace an SRAM cell’s content using current characteristics, especially the leakage current. Increasing the design density increases the leakage current; hence, SRAM cells are more prone to PA attacks.

**Figure 22:**
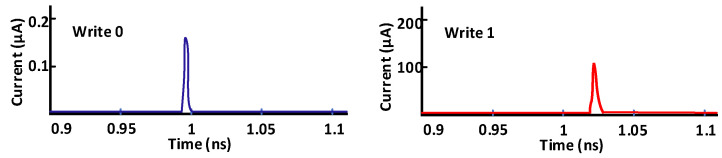
Write current flow information 6T-SRAM cell for internal data sneaking [74].

Figure 22 illustrates the concept of the PA attack by assuming an SRAM cell is storing a ‘0’. The write ‘0’ operation showed a value of about 160 nA, whereas the write ‘1’ operation showed the current value of 100 µA. Thus, a side attacker can guess internal data through the leakage of current information of a write operation. For PA, an attacker needs knowledge of and access to the internal architecture such as timing control, power distribution or peripheral circuitry.

2. Supply Voltage aging [75]: Generally, the supply voltage connection to the circuit is through PMOS devices. The aging of the gate oxide under negative biasing temperature instability (NBTI) puts the gate oxide under stress, which leads to the change in characteristics of pull-up PMOS devices in an SRAM cell. The differential change in the V_th_ of devices results in distinct power-up levels. Attackers usually perform the read operation alongside power-up level information to predict the stored data.

3. Cold Boot Attack [76]: We typically assume a volatile memory loses internal data as power turns off. However, a volatile memory holds data for quite some time after that. The retention time depends on the device material chemistry. Applying a low temperature immediately after power loss enhances retention time. Subsequent scanning of a device through external probes can reveal the stored data contents. Data encryption and cryptographic keys are among potential candidates to secure against a cold boot attack.

4. Microscopy of de-layering [77]: If an SRAM cell keeps the same data values for longer, the data imprinting effects change the transistor’s parameters. A technique known as atomic force microscopy (AFM) can provide layer information to a deeper level. Extensive analysis of information predicts data stored in the SRAM cells. The modern FinFET-SRAM cell’s lower dimensions need less area analysis which eases data stealing.

Besides data stealing, an attacker may try to fail SRAM cells. Failure attack could contain gate resizing, gate oxide thickness alteration or supply voltage variations.

**Figure 23:**
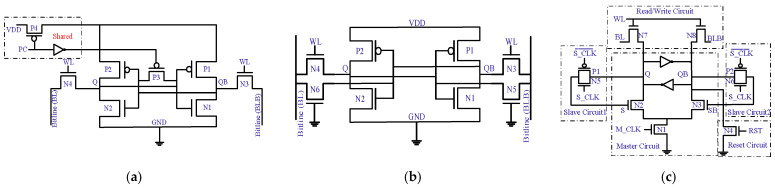
Modified SRAM cell designs for data security. (**a**) SRAM cell with balanced current before read and write operations [74]. (**b**) Incorporating false leakage current information to protect data [78]. (**c**) SRAM cell toggling values to mitigate NBTI effects [79].

B. Solutions: Power analysis and data-imprinting effects jeopardize data security, but modifications in the conventional 6T-SRAM cell mitigate them. Figure 23 presents security-aware SRAM cell designs. [74,78,79].

The SRAM cell in Figure 23a equates to the current information irrespective of a cell having a ‘0’ or ‘1’ value. Pre-charge (PC) signal goes low to connect cells to the supply voltage via transistor P4. As PC goes high, it cuts power off and turns on the P3 transistor through an inverter, equalizing the voltage level on both internal nodes, i.e., Q and QB. Any write operation will result in the same current for the ‘0’ or ‘1’ value.

Next, Figure 23b presents an 8T-SRAM cell with additional transistors N5 and N6. The gates of these transistors are tied to the ground, thus keeping them always off. Regardless of internal nodes’ values, the SRAM cell will have the same number of off transistors; so, an SRAM cell can balance out the leakage current, and this makes tracing unfeasible.

Figure 23c shows the architecture of data toggling to diminish the NBTI aftermath anytime either an N2 or N3 transistor is on as per the internal node value. Next, as the M_CLK signal goes high, a ‘0’ value is copied to Q or QB via slave circuit1 or slave circuit2 through an N2 or N3 transistor, respectively. Generally, the master circuit toggles the data values then the slave circuit copies that specific value. At the same time, the reset circuit resets the value of Q and QB to ‘1’ and ‘0’, respectively. Nevertheless, frequent data toggling reduces imprints but suffers from the hardware and power overhead.

## 8. Discussion

This section discusses prominent challenges related to the SRAM operation and its application as CIM for AI in brief.

A. Bitline voltage swing: Narrow differential voltage (around 50–200 mV) sensing ensures low read latency. Accordingly, a quick and reliable sense amplifier design that accurately detects BLs’ differential voltage [17] is challenging. Single-ended sensing schemes are power hungry; therefore Chandras et al. [80] proposed three single-ended BL-sensing schemes to optimize power consumption.

B. Biasing temperature instability: PBTI is associated with NMOS, so NBTI is related to PMOS. The root cause is electron traps (ET) in gate oxide, affecting the V_th_ of a transistor. Consequently, SRAM performance degrades or even becomes unacceptable [17]. M. Duang et al. [81] uses cyclic and anti-neutralization models with an appropriate voltage level and duration to protect against ETs.

C. Half-select SRAM cells: To access a specific SRAM cell, the row driver and column decoder select one row and column, respectively. Besides a selected cell, SRAM cells in the same row or column are selected either column-wise or row-wise, but not both. Such cells, known as half-select cells, suffer from the risk of the storage node value flipping through BLs. Internal nodes are isolated during the read and write operations and SRAM cell level alterations can make the SRAM cells half-select free [82].

D. Manufacturing and material issues: Fabrication process imperfections cause short, void, and open interconnects, referred to as hard errors. Hard fault detection and correction approaches [83] add significant overhead. Modern 3D monolithic structures for SRAM use through-silicon vias (TSV) or multiple inter-tier vias (MIV) [84] for inter-tier communication that exaggerates hard errors’ occurrence.

In addition, material losses become significant in the long run. The performance matrix, for example, means time to failure (MTTF) [85] predicts SRAM performance expectancy.

E. SRAM-based CIM: Deep neural networks (DNNs) duplicate human brain structure, enabling them to execute AI tasks with high accuracy and efficiency [86]. However, DNNs are data-centric and accelerate the data traffic between microprocessor and memory, thus becoming energy hungry. CIM performs frequent NN computations in or near memory to reduce the data traffic. SRAM-CIM for content addressable memory (CAM) [87], neuromorphic vision [88] and convolution neural networks [86] has proven SRAM as a potential candidate for AI applications.

SRAM achieves computation operations either inside or at the periphery of memory using special computational units, known as in/near computations (IMC/NMC). Experts have explored SRAM-CIM for analog or digital signal domains. Analog CIM solutions are energy efficient but suffer from non-idealities [89]. Digital CIM accelerators are free from analog signal precision and margin issues but at the cost of more peripheral circuits. Among the DNNs, multiply and accumulate (MAC) operations [89] are frequent; hence, it is focused on quite often. On-chip dedicated arithmetic units complete DNNs’ essential calculations to reduce off-chip data access and achieve performance efficiency.

## 9. Conclusions

Modern deep submicron technological nodes present design and operational hurdles to SRAMs. We have reviewed the design challenges by keeping the deep sub-micron and FinFET technologies in focus. This is important in the context of emerging CIM applications for ML and IoT devices. In this regard, we have introduced the SRAM cell configurations with performance evaluation parameters. Overall, the SRAM cell’s hurdles can be classified into five main categories. Each section highlights the mathematical parameters required to evaluate a particular challenge’s severity, followed by the potential candidates for a solution. The first two categories–the low voltage operation and leakage current–concentrate on low power operations. The next two categories–process variations and soft errors–are salient for the SRAM cell’s operational reliability. The last category is the security-sensitive design, a major concern in current systems. Alongside the generalization of multiple state-of-the-art solutions, it explains how to address design roadblocks.

Future SRAM cells should be more robust and performance-efficient to keep up with the pace of microprocessor requirements. For future research, CIM needs extensive exploration in the context of the in-memory digital domain computing as part of AI for miniaturized electronic devices. These devices are power-limited; thus, computing needs reliable and low-power operations. This study forms a foundation for the understanding of SRAM challenges before exploring SRAM-based CIM for ML.

## Figures and Tables

**Table 1:** Performance evaluation parameters for SRAM cells.

S. No.	Parameters	Explanation
1	Read Access Time	WL activation (50%) to 50–200 mV differential bitline voltage
2	Write Access Time	WL activation (50%) to 90%/10% of rising/falling internal node
3	Read/Write Power	Power consumption during read/write access time
4	Leakage Power	Power consumption during standby or hold mode
5	Cell Area	Layout area of the one SRAM cell
6	Noise Margins	SRAM noise tolerance under hold/read/write mode
7	Reliability	Performance under process/voltage/environmental variations
8	Soft Error	Critical charge accumulation to flip the internal node value

**Table 2:** Performance analysis of 6T-12T SRAM cells. (Simulation in 65 nm CMOS at nominal voltage).

S. No	Parameter	6T	7T [26]	8T [27]	9T [28]	10T [29]	11T [30]	12T [31]
1	Area Overhead *	0%	16%	30%	43%	58%	71%	89%
2	Read Dynamic Power (µW)	16.85	25.48	18.14	18.96	23.73	58.15	73.1
3	Write Dynamic Power (µW)	24.31	7.5	26.52	8.19	27.49	50.83	69.1
4	Leakage Power (nW)	5.6	5.3	5.98	6.13	5.99	2.26	7.69
5	Read Access Time (ps)	3.06	9.18	16.04	17.23	36.9	91.2	103.5
6	Write Access Time (ps)	33.53	27.96	37.93	36.7	41.83	61.06	118.3
7	Sensing Method	Differential	Single Ended	Single Ended	Single Ended	Single Ended	Single Ended	Single Ended

* Area estimation using 65 nm design rules.

**Table 3:** Summary of assist scheme for read and write at the low voltage.

S. No.	Assist Scheme	Type	Overhead
1	Negative BL (NBL)	Write	Coupling capacitor
2	Suppressed BL (SBL)	Read	Discharge devices
3	WL overdrive (WLOD)	Write	Charge pump and level shifter
4	WL under-drive (WLUD)	Read	PMOS devices and bias current
5	V_DD_ boosting	Read	Column MUX
6	V_DD_ lowering	Write	Pull-up and pull-down devices
7	G_ND_ boosting	Write	External level shifter
8	G_ND_ lowering	Read	External level shifter

**Table 4:** Summary of data attack techniques to sneak into the SRAM cell.

S. No.	Technique	Description
1	Power Analysis (PA)	Power consumption information to predict internal data
2	Supply Voltage aging	Variation in supply voltage as of NBTI in PMOS device
3	Cold Boot Attack	Data retention at lower temperatures
4	Delayering Microscopy	Data imprinting effects to guess stored data

## Data Availability

Not applicable.
